# On a new generalized symmetric vector equilibrium problem

**DOI:** 10.1186/s13660-017-1511-z

**Published:** 2017-09-22

**Authors:** Rahmatollah Lashkaripour, Ardeshir Karamian

**Affiliations:** Department of Mathematics, Faculty of Mathematics, University of Sistan and Baluchestan, Zahedan, Iran

**Keywords:** 49J40, 47H10, 47H09, KKM-mapping, nonlinear scalarization mapping, symmetric vector equilibrium problem

## Abstract

In this paper, a new form of the symmetric vector equilibrium problem is introduced and, by mixing properties of the nonlinear scalarization mapping and the maximal element lemma, an existence theorem for it is established. We show that Ky Fan’s lemma, as a usual technique for proving the existence results for equilibrium problems, implies the maximal element lemma, while it is useless for proving the main theorem of this paper. Our results can be viewed as an extension and improvement of the main results obtained by Farajzadeh (Filomat 29(9):2097-2105, 2015) and some corresponding results that appeared in this area by relaxing the lower semicontinuity, quasiconvexity on the mappings and being nontrivial of the dual cones. Finally, some examples are given to support the main results.

## Introduction and preliminaries

Existence results for vector equilibrium problems (in short, VEP) have been extensively studied in recent years. It is well-known that VEP provides a general model of several classes of problems such as the vector variational inequality, the vector complementarity problems, the vector optimization problems, the vector saddle point (minimax) problems, the multiobjective game problems, and fixed point problems (see, e.g., [2–9] and the references therein).

The symmetric vector equilibrium problem (in short, SVEP) which is a generalization of the vector equilibrium problem has been studied by many authors. One of the important symmetric vector equilibrium problems is to investigate the existence theorems in order to guarantee its solution set is nonempty (see, e.g., [10–12] and the references therein).

Recently, Farajzadeh [1] established some existence results for a solution of SVEP whose proof strongly depends on non-triviality of the dual cones, lower semicontinuity and quasiconvexity of the mappings (see Theorem 3.4 in [1]). In this paper, we first introduce a general form of SVEP (called GSVEP), and then we relax the non-triviality of the cones, lower semicontinuity and quasiconvexity that appeared in Theorem 3.4 in [1] by ‘mixing’ the properties of the nonlinear scalarization mapping and the maximal element lemma (see Lemma 2.5 and Theorem 3.1). It is worth noting that the hypotheses of lower semicontinuity and quasiconvexity on the mappings are common assumptions in most of papers that have appeared in this area.

In the rest of this section we introduce our problem and recall some notations which are needed in the next sections. Throughout the paper, unless otherwise specified, we use the following notations.

Let $I_{m} $ denote the finite index set $\lbrace1,2,\ldots,m \rbrace$. For each $i \in I_{m} $, $X_{i} $ and $Y_{i} $ stand for topological vector spaces (for short, t.v.s.) and $C_{i}$ is a proper, closed and convex cone of $Y_{i}$ with $\operatorname{int} C_{i}\neq\emptyset$, where $\operatorname{int} C_{i}$ denotes the topological interior of $C_{i}$.

The symbols $X=\prod_{i \in I_{m}} X_{i}$ and $C=\prod_{i \in I_{m}} C_{i} $ denote the Cartesian product of $X_{i}$ and $C_{i}$, respectively. So, for each $x \in X$ and $c \in C$, we have $x=(x_{i})_{i \in I_{m}}$ and $c=(c_{i})_{i \in I_{m}}$, where $x_{i}\in X_{i}$ and $c_{i}\in C_{i}$. It is well known that *X* and *C* are respectively t.v.s. and proper, closed and convex cone with $\operatorname{int} C=\prod_{i \in I_{m}} \operatorname{int} C_{i}$. The dual cone of $C_{i}$ is denoted by $C_{i}^{\ast}$ and defined by $C_{i}^{\ast}=\lbrace f\in Y_{i}^{\ast} : f(c_{i}) \geq0, \forall c_{i}\in C_{i} \rbrace$, where $Y_{i}^{\ast}$ is the topological dual space of $Y_{i}$. For each $i \in I_{m}$, let $K_{i} $ be a nonempty, closed and convex subset of $X_{i}$ and $F_{i}:\prod_{j \in I_{m} } K_{j} \times K_{i} \to 2^{Y_{i} } $ be a set-valued mapping with nonempty values, where $2^{Y_{i} }$ denotes the class of all subsets of $Y_{i} $. Now, we are ready to introduce the following problem which we call a generalized symmetric vector equilibrium problem (in short, GSVEP):

Find $(x_{j}^{*} )_{j \in I_{m}} \in\prod_{j \in I_{m} } K_{j}$ such that, for each $i \in I_{m}$,

1.1 $$ F_{i} \bigl(\bigl(x_{j}^{*} \bigr)_{j \in I_{m} },y_{i} \bigr) \cap-\operatorname{int} C_{i}= \emptyset, \quad \forall y_{i}\in K_{i}, $$

where $((x_{j}^{*} )_{j \in I_{m}},y_{i} ) =(x^{*}_{1},x^{*}_{2},\ldots,x^{*}_{m},y_{i})$.

### Remark 1.1

GSVEP is a generalization of all the following problems:

(i) If we take $I_{2}=\lbrace1,2\rbrace$, then GSVEP collapses to the symmetric vector equilibrium problem; see [1] and the references therein.
(ii) If we take $I_{2}=\lbrace1,2\rbrace$, $C_{1} = C_{2}$;
  $$f_{1}:\prod_{i \in I_{2} } K_{i} \to X_{1} $$
  and
  $$f_{2}:\prod_{i \in I_{2} } K_{i} \to X_{2} $$
  are two single-valued mappings, and
  $$F_{1}(x, y, z)=\bigl\lbrace f_{1}(z,y)-f_{1}(x, y)\bigr\rbrace ,\quad \forall \bigl((x, y), z\bigr) \in\prod _{i \in I_{2}}K_{i} \times K_{1} $$
  and
  $$F_{2}(x, y, z)=\bigl\lbrace f_{2}(x,z)-f_{2}(x, y)\bigr\rbrace ,\quad \forall \bigl((x, y), z\bigr) \in\prod _{i \in I_{2}}K_{i} \times K_{2}, $$
  then (1.1) reduces to the symmetric vector equilibrium problem which was studied in [10].
(iii) If we take $I_{2}=\lbrace1,2\rbrace$, $f_{1}: K_{1}\times K_{1}\rightarrow2^{Y_{1}}$, $F_{2}=\lbrace0_{Y_{2}} \rbrace$ and
  $$F_{1}(x , y, z)=\bigl\lbrace f_{1}(x , z)\bigr\rbrace , $$
  where $((x , y), z)\in\prod_{i\in I_{2} } K_{i} \times K_{1}$.
  Then (1.1) is the vector equilibrium problem which was introduced by Blum and Oettli [13]. For more details, we refer to [5, 14–16] and the references therein.
(iv) We can state the classical vector variational inequality problem which was introduced by Giannessi [15] in the form of GSVEP as follows:
  Take $I_{2}=\lbrace1,2\rbrace$, $T: K_{1}\rightarrow L(X_{1},X_{2})$ and define
  $$F_{1}\bigl((x_{1},x_{2}),y_{1}\bigr) = T(x_{1}) (y_{1}-x_{1}),\quad \forall \bigl((x_{1},x_{2}),y_{1}\bigr)\in\prod _{i \in I_{2} } K_{i} \times K_{1} $$
  and
  $$F_{2}=\lbrace0_{Y_{2}}\rbrace, $$
  where $L(X_{1},X_{2})$ denotes the space of all continuous linear operators from $X_{1}$ to $X_{2}$.
(v) Finally, if we take $I_{1}=\lbrace1\rbrace$, $C_{1}=[0, +\infty]$, $Y_{1}=\mathbb{R}$, $K_{1}\subseteq\mathbb{R}$, then (1.1) reduces to the scalar equilibrium problem for
  $$F_{1}: K_{1}\times K_{1}\rightarrow2^{\mathbb{R}}, $$
  which was studied by many authors (see, for example, [17, 18] and the references therein).

## Some notes on nonlinear scalarization mapping, Ky Fan’s lemma and maximal element lemma

In this section, we introduce the nonlinear scalarization mapping and some of its important properties. Also, the maximal element lemma (i.e., Lemma 2.5) and the notion of KKM mapping and Ky Fan’s lemma (i.e., Lemma 2.4) are stated. Moreover, we show that Lemma 2.5 and Ky Fan’s lemma are not equivalent. It is a remarkable fact that we cannot apply Ky Fan’s lemma, which plays an important role in the study of the existence results of equilibrium problems (see, e.g., [1, 19] and the references therein), when we work with the scalarization mapping, while Lemma 2.5 is a useful tool for proving our existence results.

The nonlinear scalarization mapping that plays a key role in the paper was first introduced in [20] in order to study the vector optimization theory and vector equilibrium problems (see [14]).

### Definition 2.1

[21, 22]

Let *X* be a topological vector space with the closed, convex and pointed cone *C*. The formula

$$\xi_{e}(x) :=\operatorname{inf} \lbrace r \in\mathbb{R}: re-x\in C \rbrace, $$

where $x \in X$ and $e \in\operatorname{int} C$, defines a mapping from *X* into $\mathbb{R}$ and is called the nonlinear scalarization mapping on *X*(with respect to *C* and *e*).

The following lemma characterizes some of the important properties of the nonlinear scalarization mapping which are used in the sequel.

### Lemma 2.2

[21, 23, 24]

*Let* *X* *be a t*.*v*.*s*. *and* *C* *be a proper*, *closed and convex cone of* *X* *with* $e\in\operatorname{int} C $. *Then*, *for each* $r \in\mathbb{R}$ *and* $x \in X$, *the following statements are satisfied*.

(i) $\xi_{e} (x) = \min \{r\in\mathbb{R}: re-x\in C \}$.
(ii) $\xi_{e} (x) \leq r \Longleftrightarrow re-x\in C $.
(iii) $\xi_{e} (x) < r \Longleftrightarrow re-x\in \operatorname{int} C $.
(iv) $\xi_{e} (x) = r \Longleftrightarrow x \in re-\partial C$, *where* *∂C* *is the topological boundary of* *C*.
(v) $y_{2}-y_{1}\in C \Longrightarrow\xi_{e}(y_{1}) \leq\xi_{e}(y_{2})$.
(vi) *The mapping* $\xi_{e} $ *is continuous*, *positively homogeneous and subadditive* (*that is*, *sublinear*) *on* *X*.

For proving an existence result of the equilibrium problems, Ky Fan’s lemma plays a key role. Now we are going to state it. Before stating it, we also need the following definition.

### Definition 2.3

[25]

Let *K* be a nonempty subset of the topological vector space *X*. A set-valued mapping $T:K\rightarrow2^{X}$ is called a KKM-mapping if, for every finite subset $\lbrace x_{1},x_{2},\ldots,x_{n}\rbrace$ of *K*, $\operatorname{conv}\lbrace x_{1},x_{2},\ldots,x_{n}\rbrace$ is contained in $\bigcup_{i=1}^{n}T(x_{i})$, where conv denotes the convex hull.

Ky Fan in 1984 obtained the following result, which is known as Ky Fan’s lemma.

### Lemma 2.4

Ky Fan-1984 [25]

*Let* *K* *be a nonempty subset of topological vector space* *X* *and* $T: K \rightarrow2^{X}$ *be a KKM mapping with closed values in* *K*. *Assume that there exists a nonempty compact convex subset* *B* *of* *K* *such that* $\bigcap_{x\in B}T(x)$ *is compact*. *Then*

$$\bigcap_{x\in K}T(x)\neq\emptyset. $$

Note that if we have a multivalued mapping *A* on a set *K* and there exists an element $x\in K$ such that $A(x)$ is empty, then, in a way, such element *x* is called ‘maximal’. The existence of maximal elements for a multivalued mapping in topological vector spaces and its important applications to mathematical economies have been studied by many authors in both mathematics and economies, see, for example, [26–28] and the references therein. Moreover, the maximal element lemma plays a crucial role in establishing the existence of solutions for GSVEP. In the following, for the sake of readers, we prove it by Ky Fan’s lemma.

### Lemma 2.5

*Let* *K* *be a nonempty convex subset of a t*.*v*.*s*. *X* *and* $A:K \longrightarrow2^{K}$ *be a set*-*valued mapping such that*

(i) *for each* $x \in K$, $A(x)$ *is convex*,
(ii) *for each* $x \in K$, $x \notin A(x)$,
(iii) *for each* $y \in K$, $A^{-1} (y)=\{x\in K : y\in A(x)\}$ *is open in* *K*,
(iv) *there exist a nonempty compact convex subset* *B* *of* *K* *and a nonempty compact subset* *N* *of* *K* *such that*
  $$A(x) \cap B \neq\emptyset,\quad \forall x\in K\backslash N. $$

*Then there exists* $x^{\ast}\in K$ *such that* $A(x^{\ast})=\emptyset$.

### Proof

Define $T:K\rightarrow2^{X} $ as follows:

$$T(x)=\bigl(A^{-1}(x)\bigr)^{c}, $$

where $x\in X$ and $(A^{-1}(x))^{c}$ denotes the complement of $A^{-1}(x)$ in *K*. By (iii), $T(x)$ is closed for each $x\in K$. We claim that *T* is a KKM mapping. To verify this, let $B=\lbrace x_{1},x_{2},\ldots,x_{n}\rbrace\subseteq K$ and $z\in \operatorname{conv}B$. If, on the contrary, we assume that $z\notin \bigcup_{i=1}^{n}(A^{-1}(x_{i}))^{c}$, then we have

$$x_{i}\in A(z),\quad \forall i=1,2,\ldots,n. $$

By (i), $z\in A(z)$ which is contradicted by (ii), and this completes the proof of the assertion. Moreover, it follows from condition (iv) that $\bigcap_{y\in B}(A^{-1}(y))^{c}\subseteq N$. Since $\bigcap_{y\in B}(A^{-1}(y))^{c}$ is a closed subset of the compact set *N*(note that the values of *T* are closed), we get that $\bigcap_{y\in B}(A^{-1}(y))^{c}$ is a compact subset of *N*, and so *T* satisfies all the assumptions of Lemma 2.4. Hence it follows from Lemma 2.4 that $\bigcap_{x\in K}T(x)\neq\emptyset$. Then there exists $x^{\ast}\in K$ such that $x^{\ast}\in\bigcap_{x\in K}T(x)$. This means

$$x\notin A\bigl(x^{\ast}\bigr),\quad \forall x\in K. $$

Hence $A(x^{\ast})=\emptyset$. This completes the proof. □

### Remark 2.6

By reviewing the proof of Lemma 2.5, one can replace conditions (i) and (ii) by the following condition:

$$x \notin \operatorname{conv}A(x),\quad \forall x \in K. $$

Because in line seven of the proof we obtained

$$x_{i}\in A(z),\quad \forall i=1,2,\ldots,n, $$

where $z=\sum_{i = 1}^{n} \lambda_{i}x_{i}$, $\sum_{i = 1}^{n} \lambda_{i}=1$ and $\lambda_{i}\geq0$ for all *i*, so

$$x_{i}\in \operatorname{conv}A(z). $$

Hence $z=\sum_{i = 1}^{n} \lambda_{i}x_{i}\in \operatorname{conv}A(z) $ and the rest of the proof can be continued.

Moreover, it follows from condition (ii) that the set-valued mapping *A* is never a KKM mapping. Hence we cannot use Ky Fan’s lemma for mapping *A*.

The following lemma shows that Lemma 2.5 implies Fan’s lemma (Lemma 2.4) when the mapping $T:K\rightarrow2^{X} $ has the following property:

$$T\bigl(\lambda x+(1-\lambda) y\bigr)\subseteq Tx\cup Ty,\quad \forall(x,y, \lambda )\in K\times K\times[0,1]. $$

### Lemma 2.7

*Let* *K* *be a nonempty subset of a topological vector space* *X* *and* $T: K \rightarrow2^{X}$ *be a mapping with closed values in* *K*. *Assume that there exists a nonempty compact convex subset* *B* *of* *K* *such that* $\bigcap_{x\in B}T(x)$ *is compact*. *In addition*, *we assume that* $x\in Tx$ *for each* $x\in K$ *and*

$$T\bigl(\lambda x+(1-\lambda) y\bigr)\subseteq Tx\cup Ty,\quad \forall(x,y, \lambda )\in K\times K\times[0,1]. $$

*Then* $\bigcap_{x\in K}T(x)\neq\emptyset$.

### Proof

It is clear that

$$T\bigl(\lambda x+(1-\lambda) y\bigr)\subseteq Tx\cup Ty\quad \Longleftrightarrow\quad A(x)=K \smallsetminus T^{-1}x \quad \mbox{is convex}, $$

where $(x,y,\lambda)\in K\times K\times[0,1]$.

Now, by letting $N=\bigcap_{x\in B}T(x)$, one can check the mapping

$$A:K\rightarrow2^{K} $$

defined by

$$A(x)=K \smallsetminus T^{-1}x $$

satisfies all the assumptions of Lemma 2.5. Thus there exists $x^{\ast}\in K$ such that $A(x^{\ast})=\emptyset$.

Therefore, from the definition of *A*, we get $x^{\ast}\in Tx$, $\forall x\in K$ and so

$$\bigcap_{x\in K}T(x)\neq\emptyset. $$

 □

The following example illustrates Lemma 2.7.

### Example 2.8

Let $X=\mathbb{R}$, $K=[0,1]$ and define $T:K\longrightarrow2^{X}$ by $Tx=[x,1]$. It is easy to check that *T* satisfies all the conditions of Lemma 2.7 and $\bigcap_{x\in K}T(x)=\{1\}$.

The following lemma provides a link between a vector ordering and a scalar ordering, which is a useful tool for reducing a vector problem to the scalar problem.

### Lemma 2.9

*Let* $\lbrace Y_{i}\rbrace_{i\in I_{m}}$ *be a family of t*.*v*.*s*., $\mathbb {R}_{+}^{m}=\lbrace(t_{i})_{i\in I_{m}} : t_{i}\geq0, \forall i\in I_{m}\rbrace$ *and* $e=(e_{i} )_{i \in I_{m}} \in\prod_{i \in I_{m}}\operatorname{int} C_{i} $ *with* $C_{i} \subseteq Y_{i} $. *Then*, *for any* $B_{i}\subseteq Y_{i}$, *we have*

$$B_{i}\cap-\operatorname{int} C_{i}=\emptyset,\quad \forall i\in I_{m}\quad \Longleftrightarrow\quad P_{e}\biggl(\prod _{i \in I_{m}} B_{i}\biggr)\subseteq\mathbb{R}_{+}^{m}, $$

*where* $P_{e}(\prod_{i \in I_{m}} B_{i})=\prod_{i \in I_{m}} \xi_{e_{i} }(B_{i} )$ *and* $\xi_{e_{i} }(B_{i} ) $ *is the image of* $B_{i}$ *under* $\xi _{e_{i} }$.

### Proof

It follows from Lemma 2.2(iii) that

$$\begin{aligned} B_{i}\cap-\operatorname{int} C_{i}=\emptyset,\quad \forall i\in I_{m}&\quad \Longleftrightarrow\quad \xi_{e_{i}}(x)\geq0,\quad \forall x \in B_{i},\forall i\in I_{m} \\ &\quad \Longleftrightarrow\quad \xi_{e_{i}}(x_{i})\geq0,\quad \forall i\in I_{m}. \end{aligned}$$

Hence the result follows. □

To prove the main results of this paper, we need the following lemma.

### Lemma 2.10

*Let* $\lbrace X_{i}\rbrace_{i\in I_{m}}$ *be a family of t*.*v*.*s*. *For any* $i \in I_{m}$, *let* $K_{i} \subseteq X_{i} $ *be a nonempty closed convex set*, $x^{\ast}=(x_{j}^{*} )_{j \in I_{m}} \in\prod_{j \in I_{m} } K_{j}$ *and* $P_{e}$ *be as given in Lemma* 2.9. *Then* $x^{\ast}$ *is a solution of* (1.1) *if and only if*

$$ P_{e} \biggl(\prod_{i\in I_{m}} F_{i} \bigl(\bigl(x_{j}^{*} \bigr)_{j \in I_{m} },y_{i} \bigr) \biggr) \subseteq\mathbb{R}_{+}^{m},\quad \forall(y_{i} )_{i \in I_{m} } \in\prod_{j \in I_{m}} K_{j}. $$

### Proof

It is sufficient, for each $i\in I_{m}$, we take $B_{i}=F_{i}((x_{j}^{*} )_{j \in I_{m} },y_{i} ) $. Now, the result follows by Lemma 2.9. □

## Existence results

Now, we are ready to present an existence result of a solution for GSVEP by using the scalarization method and the maximal element lemma, which one can consider as an extension of the well-known results in this area from SVEP to GSVEP. In fact, some sufficient conditions to guarantee the existence of the solution for GSVEP, by relaxing the lower semicontinuity, quasiconvexity on the mappings and without assuming the non-triviality (that is, each cone contains a nonzero element) of the dual cones of the spaces, which in most of references are assumed to be nontrivial, are given. Moreover, in order to illustrate the main theorem of this section, some examples are provided. We note that Ky Fan’s lemma is useless for proving Theorem 3.1 (see Remark 2.6).

### Theorem 3.1

*Let* $\lbrace X_{i}\rbrace_{i\in I_{m}}$ *and* $\lbrace Y_{i}\rbrace_{i \in I_{m}}$ *be two families of t*.*v*.*s*. *For each* $i\in I_{m}$, *let* $K_{i} $ *be a nonempty closed convex subset of* $X_{i}$, $C_{i}$ *be a proper*, *closed and convex cone in* $Y_{i}$, $P_{e}$ *be the same as in Lemma* 2.9 *and* $F_{i}:\prod_{j \in I_{m}} K_{j} \times K_{i} \longrightarrow 2^{Y_{i}}$ *be a set*-*valued mapping with nonempty values*. *Assume that the following conditions hold*:

(i) *for all* $(x_{j})_{j \in I_{m}}\in\prod_{j \in I_{m}} K_{j} $, $F_{i} ((x_{j})_{j \in I_{m}},x_{i} ) \cap-\operatorname{int} C_{i}=\emptyset$, $\forall i\in I_{m}$;
(ii) *for all* $(x_{j})_{j\in I_{m}} \in\prod_{j\in I_{m}}K_{j}$, *the set*
  $$\biggl\lbrace (y_{i})_{i\in I_{m}} \in\prod _{j \in I_{m}} K_{j}: \prod_{i\in I_{m}}F_{i} \bigl((x_{j})_{j\in I_{m}},y_{i}\bigr)\cap\prod _{i\in I_{m}}(-\operatorname{int} C_{i})\neq{\varPhi} \biggr\rbrace , $$
  *is convex*, *where* $\varPhi=\underbrace{(\emptyset,\emptyset, \ldots,\emptyset)}_{m \textit{ elements}}$;
(iii) *for all* $(y_{i})_{i\in I_{m}} \in\prod_{j \in I_{m}} K_{j}$, *the set*
  $$\biggl\lbrace (x_{j})_{j\in I_{m}} \in\prod _{j \in I_{m}} K_{j}: \prod_{i\in I_{m}}F_{i} \bigl((x_{j})_{j\in I_{m}},y_{i}\bigr)\cap\prod _{i\in I_{m}}(-\operatorname{int} C_{i})\neq{\varPhi} \biggr\rbrace ,$$
  *is open*;
(iv) *there exist a nonempty compact convex subset* $\prod_{j \in I_{m} } B_{j}$ *of* $\prod_{j \in I_{m} } K_{j} $ *and a nonempty compact subset* $\prod_{j \in I_{m} } N_{j}$ *of* $\prod_{j \in I_{m} } K_{j}$ *such that for each*
  $$(x_{j} )_{j \in I_{m}} \in\prod_{j \in I_{m} } K_{j}\smallsetminus\prod_{j \in I_{m} } N_{j}, $$
  *there exists* $(y_{i} )_{i \in I_{m}} \in\prod_{j \in I_{m}} B_{j}$ *satisfying*
  $$P_{e} \biggl(\prod_{i \in I_{m}} F_{i} \bigl((x_{j} )_{j \in I_{m}},y_{i}\bigr)\biggr)\nsubseteq \mathbb{R}_{+}^{m}. $$

*Then the solution set of* GSVEP *is nonempty*. *Moreover*, *if for all* $(y_{i})_{i\in I_{m}} \in\prod_{j \in I_{m}} K_{j}$, *the set*

$$\biggl\lbrace (x_{j})_{j \in I_{m}}\in\prod _{j \in I_{m}} K_{j} : P_{e} \biggl(\prod _{i \in I_{m}} F_{i} \bigl((x_{j} )_{j \in I_{m}},y_{i}\bigr)\biggr)\subseteq\mathbb {R}_{+}^{m}\biggr\rbrace $$

*is convex*, *the solution set of* GSVEP *is convex*.

### Proof

Let $A: \prod_{j \in I_{m}}K_{j} \longrightarrow2^{\prod_{j \in I_{m}}K_{j}} $ be defined by

$$ A\bigl((x_{j})_{j \in I_{m}} \bigr)= \biggl\{ (y_{i} )_{i \in I_{m}} \in\prod_{i \in I_{m}} K_{i} : P_{e} \biggl(\prod_{i \in I_{m}} F_{i} \bigl((x_{j} )_{j \in I_{m}},y_{i}\bigr)\biggr)\nsubseteq \mathbb{R}_{+}^{m} \biggr\} , $$

where $(x_{j} )_{j \in I_{m}}\in\prod_{j \in I_{m}}K_{j}$.

We claim that the mapping *A* satisfies all the conditions of Lemma 2.5.

First, applying Lemma 2.9 and condition (ii), it is clear that $A((x_{j})_{j \in I_{m}} )$ is a convex set for any $(x_{j})_{j\in I_{m}}\in\prod_{j \in I_{m}}K_{j}$.

Now, we show that $(x_{j})_{j\in I_{m} }\notin A((x_{j})_{j\in I_{m}})$ for any $(x_{j})_{j\in I_{m}}\in\prod_{j\in I_{m}}K_{j}$. It follows from condition (i) and Lemma 2.9 that

$$ P_{e} \biggl(\prod_{i\in I_{m}} F_{i} \bigl((x_{j} )_{j \in I_{m} },x_{i} \bigr) \biggr) \subseteq \mathbb{R}_{+}^{m}, $$

that is,

$$ (x_{j})_{j\in I_{m} }\notin A\bigl((x_{j})_{j\in I_{m}} \bigr). $$

Moreover, we claim that $A^{-1}((y_{i} )_{i \in I_{m}} )$ is open. Indeed,

$$\begin{aligned} A^{-1}\bigl((y_{i} )_{i \in I_{m}}\bigr)&=\biggl\lbrace (x_{j})_{j\in I_{m}} \in\prod_{j \in I_{m}} K_{j} : (y_{i})_{i\in I_{m}}\in A (x_{j})_{j\in I_{m}} \biggr\rbrace \\ &=\biggl\lbrace (x_{j})_{j\in I_{m}} \in\prod _{j \in I_{m}} K_{j}: P_{e} \biggl(\prod _{i \in I_{m}} F_{i} \bigl((x_{j} )_{j \in I_{m}},y_{i}\bigr)\biggr)\nsubseteq\mathbb{R}_{+}^{m} \biggr\rbrace \\ &=\biggl\lbrace (x_{j})_{j\in I_{m}} \in\prod _{j \in I_{m}} K_{j}: \prod_{i\in I_{m}}F_{i} \bigl((x_{j})_{j\in I_{m}},y_{i}\bigr)\cap\prod _{i\in I_{m}}(-\operatorname{int} C_{i})\neq{\varPhi} \biggr\rbrace , \end{aligned}$$

which is an open set by (iii).

Finally, applying condition (iv), there exist a nonempty compact convex subset $B=\prod_{j \in I_{m}} B_{j}$ of $\prod_{j\in I_{m}}K_{j}$ and a nonempty compact subset $N=\prod_{j \in I_{m} } N_{j}$ of $\prod_{j \in I_{m} } K_{j} $ such that, for any $(x_{j} )_{j \in I_{m}} \in\prod_{j \in I_{m} } K_{j}\smallsetminus N$, $(y_{i} )_{i \in I_{m}} \in A((x_{j})_{j \in I_{m}} ) \cap B $.

Thus all the conditions of Lemma 2.5 are satisfied, and then there exists $(x_{j}^{\ast})_{j\in I_{m}} \in\prod_{j \in I_{m}} K_{j}$ such that

$$A\bigl(\bigl(x_{j}^{\ast}\bigr)_{j\in I_{m}}\bigr)= \emptyset. $$

This means that for all $(y_{i})_{i\in I_{m}} \in\prod_{i \in I_{m}} K_{i}$,

$$P_{e} \biggl(\prod_{i\in I_{m}} F_{i} \bigl(\bigl(x_{j}^{\ast}\bigr)_{j\in I_{m} },y_{i} \bigr) \biggr) \subseteq\mathbb{R}_{+}^{m}. $$

Then, applying Lemma 2.10, $(x_{i}^{\ast})_{i \in I_{m}}$ is a solution of *GSVEP*. By latest condition, it is straightforward to see that the solution set of *GSVEP* is convex. This completes the proof. □

We remark that the solution set of GSVEP is convex when for all $(y_{i})_{i\in I_{m}} \in\prod_{j \in I_{m}} K_{j}$, the set

$$\biggl\lbrace (x_{j})_{j\in I_{m}} \in\prod _{j \in I_{m}} K_{j}: \prod_{i\in I_{m}}F_{i} \bigl((x_{j})_{j\in I_{m}},y_{i}\bigr)\cap\prod _{i\in I_{m}}(-\operatorname{int} C_{i})={\varPhi} \biggr\rbrace ,$$

is convex. It is easy to see that this condition is an equivalent form of the condition that appeared in Theorem 3.1 for the convexity of the solution set of GSVEP. Also, condition (iii) of Theorem 3.1 is equivalent to the following condition:

(iii)′: for all $(y_{i})_{i\in I_{m}} \in\prod_{j \in I_{m}} K_{j}$, the set
  $$\bigcup_{i=1}^{m}\biggl\lbrace (x_{j})_{j\in I_{m}} \in\prod_{j \in I_{m}} K_{j}: F_{i}\bigl((x_{j})_{j\in I_{m}},y_{i} \bigr)\cap-\operatorname{int} C_{i}\neq \emptyset\biggr\rbrace ,$$
  is open.

The following example satisfies all the assumptions of Theorem 3.1, while $F_{1}$ is not lower semicontinuous at $(\frac{1}{2}, \frac{1}{2},1)$. Because, if we take the open set $U=(1,\frac {3}{2})$, then $(F_{1}(\frac{1}{2}, \frac{1}{2},1)=[\frac {-1}{2},\frac{3}{2}])\cap U\neq\emptyset$. Now, for each neighborhood *V* of $(\frac{1}{2}, \frac{1}{2},1)$, we have $F_{1}(w_{1},w_{2},w_{3})\cap U=\emptyset$, where $(w_{1},w_{2},w_{3})\in V$ and $w_{1}\in Q^{c}$, $w_{1}<\frac{1}{2}$, $1< w_{2}$. This means that $F_{1}$ is not lower semicontinuous at $(\frac{1}{2}, \frac{1}{2},1)$. Hence it does not satisfy all the assumptions of Theorem 3.4 of [1]. Thus Theorem 3.4 of [1] does not work for the example.

### Example 3.2

Let $I_{2}=\lbrace1,2 \rbrace$, $X_{1}=X_{2}=Y_{1}=Y_{2}=\mathbb{R}$, $C_{1}=C_{2}=\mathbb{R}_{+}=\lbrace x: x\geq0\rbrace$, $e=(1,1)$, and $K_{1}=K_{2 }=[0,1]$. Let

$$q(x)= \textstyle\begin{cases} x+1, & x\in Q \cap[0,1] , \\ x ,& x\in Q^{c}\cap[0,1]. \end{cases} $$

Define the mappings $F_{1}: (K_{1}\times K_{2})\times K_{1}\longrightarrow2^{\mathbb{R}}$ and

$$F_{2}: (K_{1}\times K_{2})\times K_{2}\longrightarrow2^{\mathbb{R}} $$

by

$$F_{1}\bigl((x_{1},x_{2}),z_{1} \bigr)=\bigl[x_{1}-z_{1}, q(x_{1})\bigr], $$

and

$$F_{2}\bigl((x_{1},x_{2}),z_{2} \bigr)=[x_{2}-z_{2}, 3]. $$

We prove that the mappings $F_{1}$ and $F_{2}$ fulfill the conditions of Theorem 3.1.

Condition (i) trivially holds.

To verify condition (ii), let $(x_{1}, x_{2})\in[0,1]\times[0,1]$, $(w_{1},w_{2}), (r_{1},r_{2})\in D$ and

$$D=\bigl\{ (y_{1},y_{2}) :F_{1}(x_{1},x_{2},y_{1}) \times F_{2}(x_{1},x_{2},y_{2})\cap(- \infty,0)\times(-\infty,0) \neq (\emptyset, \emptyset) \bigr\} . $$

We show that $\lambda(w_{1},w_{2})+(1-\lambda)(r_{1},r_{2})\in D$, where $\lambda\in(0,1)$. It follows from $(w_{1},w_{2}), (r_{1},r_{2})\in D$ that

$$w_{1}>x_{1},\qquad w_{2}>x_{2} $$

and

$$r_{1}>x_{1},\qquad r_{2}>x_{2}. $$

Thus

$$\lambda w_{1}+(1-\lambda)r_{1}>x_{1} $$

and

$$\lambda w_{2}+(1-\lambda)r_{2}>x_{2}. $$

That is, assumption (ii) also holds.

To see condition (iii), let $(y_{1},y_{2})\in[0,1]\times[0,1]$ and

$$O_{i}=\bigl\{ (x_{1},x_{2})\in[0,1]\times[0,1]: F_{i}(x_{1},x_{2},y_{i})\cap(- \infty,0)\neq\emptyset\bigr\} ,\quad \forall i=1,2. $$

It is obvious that the sets $O_{1}$ and $O_{2}$ are open, condition (iii) directly follows from the equivalent condition (iii)′.

Finally, to show condition (iv), it is enough to take

$$B_{i}=N_{i}=K_{i}=[0,1],\quad \forall i=1,2. $$

Hence, applying Theorem 3.1, GSVEP has a solution. We can see that $(x_{1}^{\ast}, x_{2}^{\ast})=(1,1)$ is a solution of GSVEP. In other words, for each $i \in\lbrace1,2\rbrace$, we have

$$F_{i}\bigl((1,1),z_{i} \bigr) \cap-\operatorname{int} \mathbb {R}_{+}= \emptyset,\quad \forall z_{i} \in K_{i} . $$

### Remark 3.3

Obviously, when $I_{m}=\lbrace1,2\rbrace$, conditions (i) and (ii) in Theorem 3.1 are weaker than conditions (i) and (ii) in Theorem 3.4 in [1]. Moreover, the proof of Theorem 3.1 is different from the proof of Theorem 3.4 given in [1]. Furthermore, in Theorem 3.4 in [1] the proof is strongly dependent on the non-triviality of the dual cones, while it has been relaxed in Theorem 3.1. There are many cones whose duals are trivial, for instance, see the following example. Hence we cannot apply Theorem 3.4 in [1] for it, while Theorem 3.1 enables us to discuss the existence of solution for it.

### Example 3.4

For $0< p<1$, let $L^{p}[0,1]$ denote the set of all measurable functions $f:[0,1]\longrightarrow\mathbb{R}$ with $\int_{0}^{1} \vert f(x) \vert ^{p}<\infty$. It can be shown that the topological dual of $L^{p}[0,1]$, i.e.,$(L^{p}[0,1])^{\ast}$, equals zero. So there is no cone in $(L^{p}[0,1])^{\ast}$ containing a nonzero element.

## Conclusion

In this paper, first a new form of the symmetric vector equilibrium problem is introduced and the relationship between the maximal element lemma and Ky Fan’s lemma is discussed. Then, by mixing the properties of the nonlinear scalarization mapping and the maximal element lemma, an existence theorem for the generalized symmetric vector equilibrium problem (GSVEP), by relaxing or weakening some assumptions, is established. Moreover, under some suitable assumptions, the convexity of the solution set of GSVEP is studied. Finally, some examples are given in order to support the main results. The main results of this note can be viewed as an extension and improvement of the main results obtained by Farajzadeh (Filomat 29(9):2097-2105, 2015) and some corresponding results that appeared in this area.
